# Factors Associated with Frailty Status in Relatively Healthy Community-Dwelling Older Adults

**DOI:** 10.1093/geroni/igaa057.1579

**Published:** 2020-12-16

**Authors:** A R M Saifuddin Ekram, Joanne Ryan, Sara Espinoza, Anne Murray, Michael Ernst, Robyn Woods

**Affiliations:** 1 ASPREE, Monash University, Prahran, Victoria, Australia; 2 School of Public Health and Preventive Medicine, Prahran, Victoria, Australia; 3 University of Texas Health Science Center at San Antonio, San Antonio, Texas, United States; 4 University of Minnesota, Minneapolis, Minnesota, United States; 5 College of Pharmacy, The University of Iowa, Iowa City, Iowa, United States; 6 Monash School of Public Health and Preventive Medicine, Prahran, Victoria, Australia

## Abstract

Frailty is gaining importance as a predictor of disability and mortality in older people, and becoming frail poses a challenge for healthy aging. We investigated the prevalence and factors associated with pre-frail and frail status in a large study cohort of community-dwelling healthy older adults from Australia and the United States. A total of 19,114 individuals (87% Australian and 56% women) aged 65 years or older enrolled in a primary prevention clinical trial were evaluated. Frailty status was classified using the modified Fried phenotype criteria comprising of exhaustion, body mass index, grip strength, gait speed and physical activity. Prevalence and factors associated with frailty status (e.g. demographic characteristics and lifestyle factors) were reported using descriptive statistics along with a logistic regression model. At baseline, 2.3 % (95% CI, 2.1-2.5) of older trial participants were frail and 39.2% (95% CI, 38.5-39.9) were pre-frail, respectively. Women were more likely to be frail (65.1% vs 36.9%) and prefrail (58.0% vs 42.0%) than men. Lower levels of education (<12 years), living alone, ethnic minorities, current smoking and past alcohol use were some of the factors which were common among frail or prefrail. Despite being a relatively healthy cohort, more than one-third of the older trial participants were pre-frail, which was more prevalent among specific subgroups of individuals. This study emphasizes the high burden of the prefrailty status even among an apparently healthy cohort of community-dwelling older people.

